# *ZNF384* rearrangement in acute lymphocytic leukemia with renal involvement as the first manifestation is associated with a poor prognosis: a case report

**DOI:** 10.1186/s13039-022-00583-4

**Published:** 2022-02-14

**Authors:** Jinlong Ma, Jiaheng Guan, Baoan Chen

**Affiliations:** Department of Hematology and Oncology, School of Medicine, Zhongda Hospital, Southeast University, Nanjing, 210009 Jiangsu China

**Keywords:** Acute lymphocytic leukemia, *ZNF384*, Gene rearrangement, Immunophenotype, Prognosis

## Abstract

**Background:**

Novel fusion genes such as *ZNF384*, have been identified in B-cell precursor acute lymphoblastic leukemia (BCP-ALL) in recent years. Patients harboring *ZNF384* rearrangements have a distinctive immunophenotype with weak CD10 and aberrant CD13 and/or CD33 expression. Thus, *ZNF384*-rearranged ALL is a unique subtype of BCP-ALL. However, research on the prognostic significance of *ZNF384* rearrangements has been limited to date, especially in adolescents.

**Case presentation:**

We described a 17-year-old adolescent who was diagnosed with ALL and had renal involvement as the first manifestation, which was very rare in the existing studies. FISH analysis indicated a rearrangement of *ZNF384* according to its probe. The patient had a typical characteristic immunophenotype of *ZNF384* rearrangement, with CD10 negativity and CD13 and CD33 positivity. She had an unfavorable prognosis because she responded poorly to chemotherapy and developed a relapse shortly after reaching CR.

**Conclusion:**

The importance of *ZNF384* rearrangements in terms of prognosis remains unclear. We reported an adolescent who was diagnosed with *ZNF384*-rearranged ALL with renal involvement. She underwent different therapies, but her prognosis remained poor. Since *ZNF384* rearrangements may act as a prognostic predictor in children or adolescents, early detection based on its characteristic immunophenotype is of great necessity.

**Supplementary Information:**

The online version contains supplementary material available at 10.1186/s13039-022-00583-4.

## Background

Acute lymphocytic leukemia (ALL) is a malignant neoplasm in which the differentiation of lymphoid cells is blocked at an early stage, and there is extensive infiltration in the bone marrow, peripheral blood, and other organs. Over 80% of ALL cases are B-cell precursor acute lymphoblastic leukemia (BCP-ALL). In 2020, 6,150 new cases of ALL were diagnosed in the United States, accounting for 0.3% of all new cancer cases [1]. ALL usually occurs in children and young adults, with the peak age ranging from 2 to 5 [2].

Gene rearrangements and fusions are common in BCP-ALL, and they play an important role in determining therapeutic targets and predicting prognosis. With the advancement of genome and transcriptome sequencing, novel gene fusions, such as zinc-finger protein 384 (*ZNF384*) rearrangements have been found. The *ZNF384* gene is located on chromosome 12p13. It encodes a zinc finger protein that functions as a transcription factor and regulates the expression of matrix metalloproteinases [3]. In *ZNF384*-rearranged BCP-ALL, the breakpoints of *ZNF384* are typically located in exons 2 and 3, which contain the entire *ZNF384* protein that may be responsible for the characteristics of the immunophenotype [4, 5]. Therefore, the presence of *ZNF384* rearrangements should be a hallmark and the diagnostic criterion of a separate subtype of BCP-ALL since patients harboring such rearrangements have a distinctive immunophenotype. This immunophenotype includes weak CD10 and aberrant CD13 and/or CD33 expression (6).

Recent data have shown that as many as 1% to 6% of children and 5% to 15% of adults with BCP-ALL harbor *ZNF384* rearrangements [7]. Although the incidence is not low, such rearrangements are difficult to detect by conventional karyotype analysis, and there has been a lack of research on the prognosis of this rearrangement for a long time. Furthermore, the liver, spleen, and lymph nodes are the most common sites of extramedullary involvement in ALL, with renal involvement being relatively uncommon. The unique immunophenotype of *ZNF384* rearrangements helps to diagnose BCP-ALL patients with rare sites of extramedullary involvement. Herein, we report an adolescent case of *ZNF384*-rearranged ALL with renal involvement as the first manifestation and poor prognosis.

## Case presentation

A 17-year-old female was admitted to our hospital in December 2019 because of increased urine foam and fatigue for 3 weeks.

**Fig. 1:**
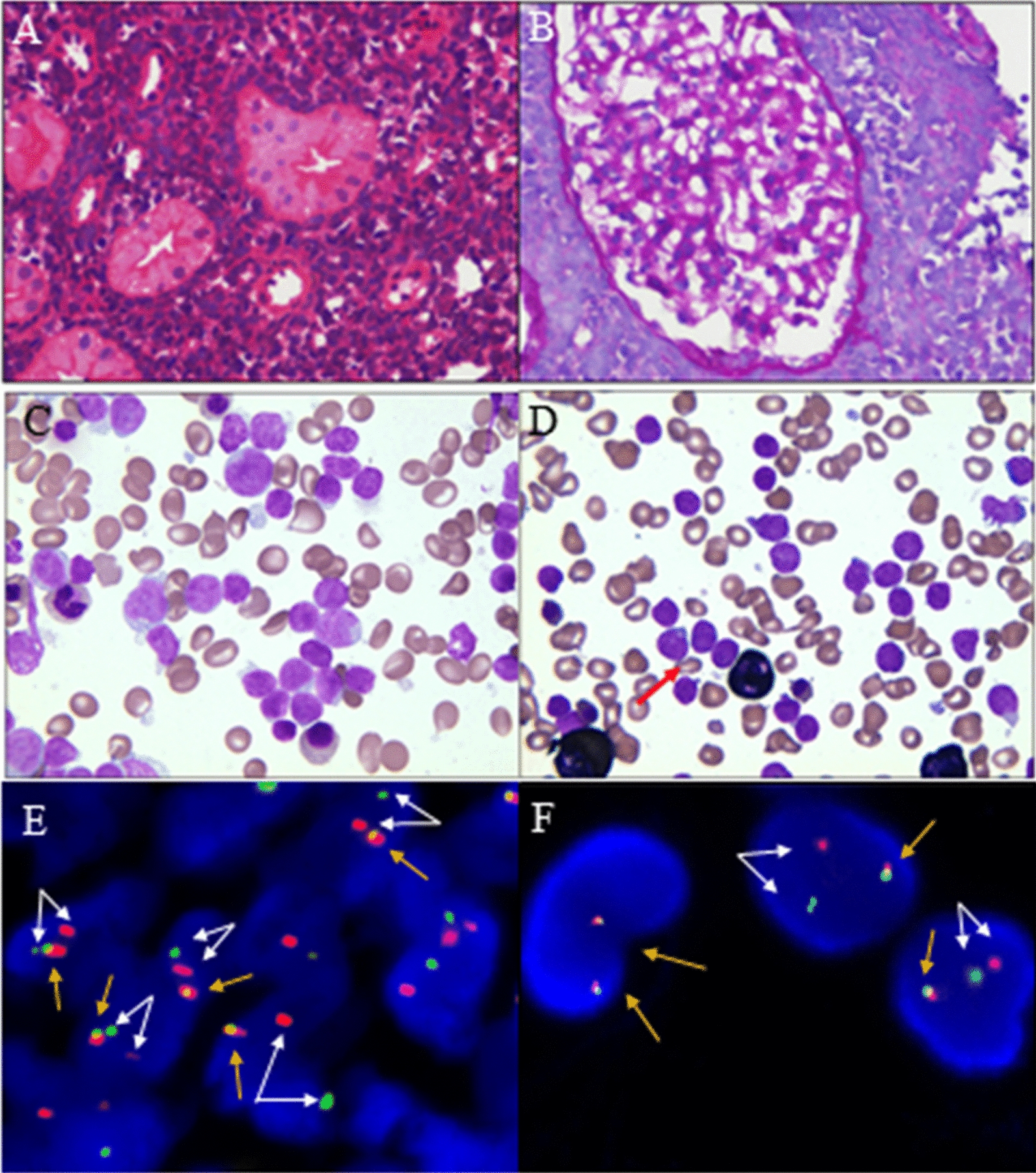
Pathological results of kidney biopsy and bone marrow aspiration smear and results of FISH. **A**, **B** The result of kidney biopsy shows increased lymphocytes, indicating lymphoblastic lymphoma or leukemia with kidney involvement (**A**: HE staining; original magnification, ×400 and **B**: PAS staining; original magnification, ×400). **C** The result of Wright-Giemsa staining of the bone marrow smear sample is shown. Bone marrow hyperplasia is extremely active, with lymphoblasts accounting for 50.8% of cells before treatment (Wright-Giemsa staining; original magnification, ×1000). **D** Lymphoblasts were negative for myeloperoxidase (red arrow), suggesting the possible diagnosis of acute lymphocytic leukemia (POX staining; original magnification, ×1000). **E** Fluorescence in situ hybridization (FISH) using a *ZNF384* break-apart probe shows *ZNF384* rearrangement in the kidney biopsy sample. Recognizable split signals can be seen in the scattered individual cells. The white arrows represent a single red signal and a single green signal (1R1G), which are split signals. The yellow arrow represents a single yellow signal (1Y), which is a normal signal. 1R1G1Y is a positive signal model for *ZNF384* rearrangement. **F** Describes the *ZNF384* rearrangement (39%) in the bone marrow sample, indicating homology with the rearrangement of *ZNF384* in the kidney biopsy sample. The two yellow arrows on the left represent two yellow signals, which are negative signals for *ZNF384* rearrangement. The white arrows and the yellow arrows on the right are indicative of *ZNF384* gene rearrangement (1R1G1Y)

**Fig. 2:**
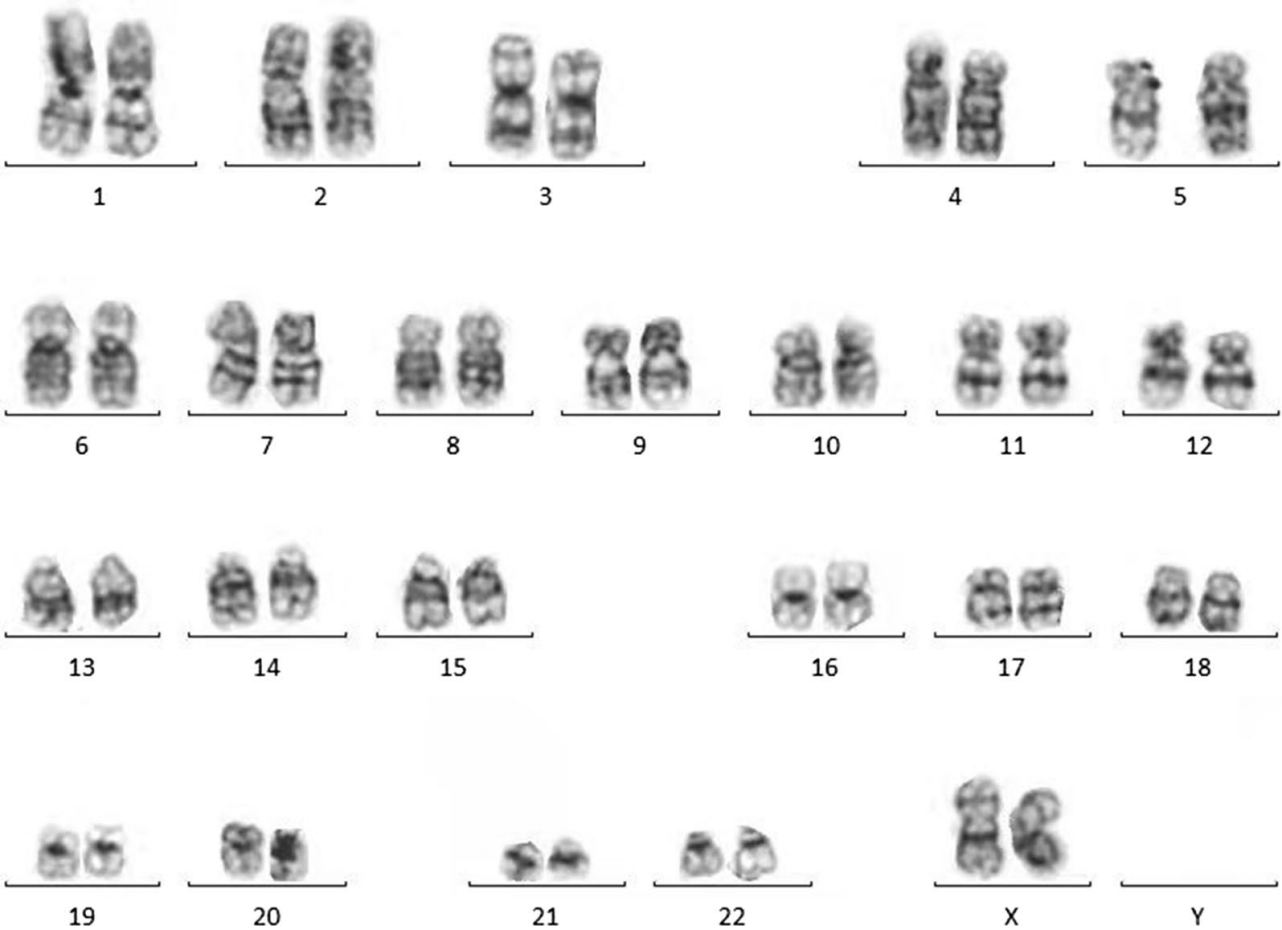
Karyotype analysis of chromosomal G-banding. The G-banded karyotype of bone marrow cells showed a normal karyotype upon admission to our hospital

**Fig. 3:**
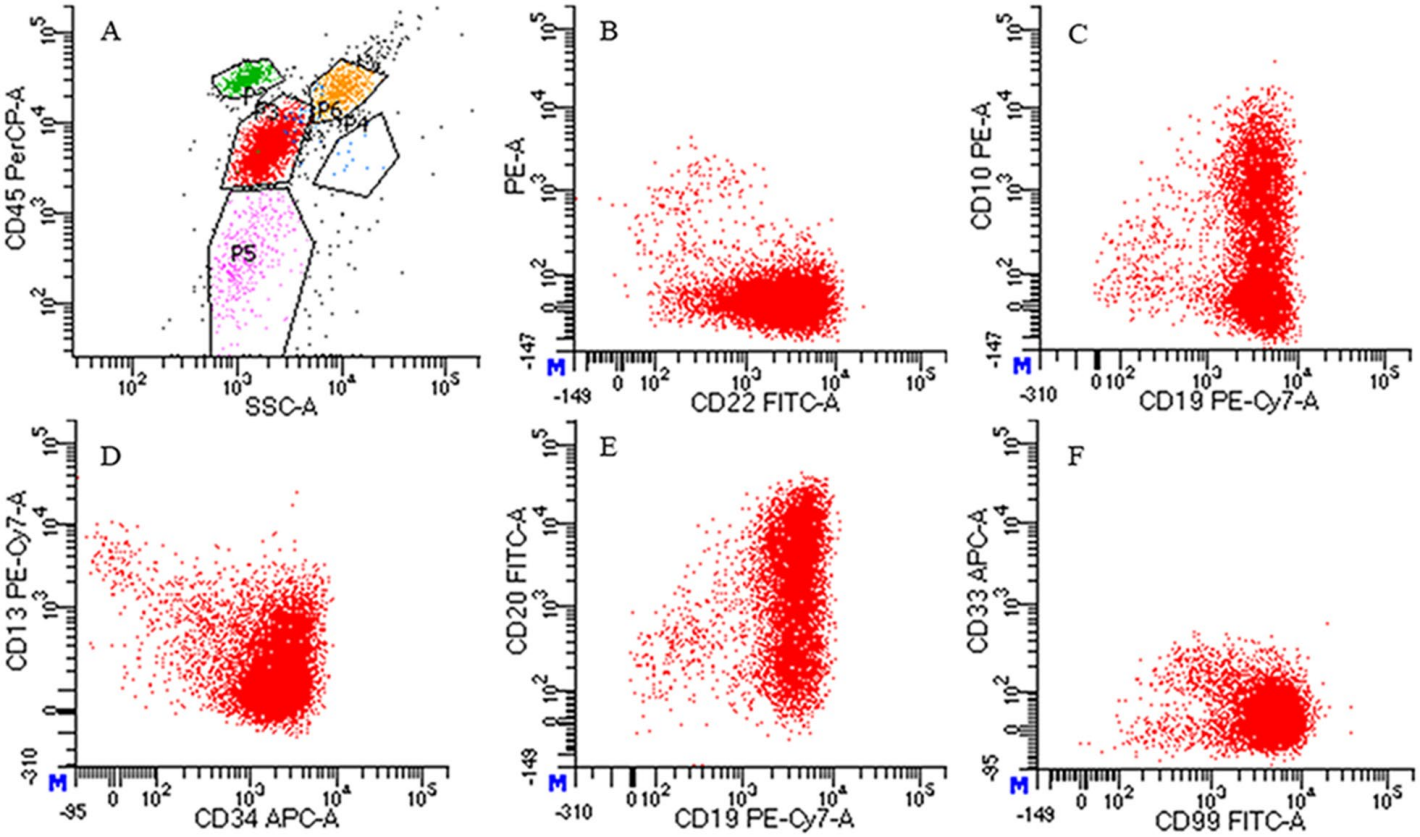
The results of flow cytometry. The flow cytometry results indicated acute lymphocytic leukemia. **A** The proportion of primary cells was 68.45% according to the SSC/CD45 gating. **B** The CD22 fraction is shown as 22.8%. **C** CD19 was positive, with a proportion of 81.1%, and CD10 was positive, with a proportion of 20.9%. **D** CD34 was positive, with a proportion of 98.2%, and CD13 was positive, with a proportion of 55.8%. **E** CD20 was positive, with a proportion of 35.0%. **F** CD33 was positive, with a percentage of 69.8%

Laboratory examinations showed protein (3+) and occult blood (2+) in urine. The blood urea nitrogen was 5.39 mmol/l, the serum creatinine was 69 μmol/l, and the lymphocyte ratio was 62.01%. Kidney color Doppler ultrasound showed increased volume in both kidneys as well as enhanced parenchymal echo. Then kidney biopsy was performed, indicating lymphoblastic lymphoma or leukemia with kidney involvement (Fig. 1A and B). Immunohistochemistry showed TdT(+), CD99(+), CD3(−), CD20(part+), CD73(−), PAX5(+), and LCA(part+). Fluorescence in situ hybridization (FISH) of the kidney biopsy sample indicated *ZNF384* rearrangement (Fig. 1E). Then, bone marrow aspiration and biopsy were performed, revealing the disappearance of fat vacuoles and the appearance of immature lymphoid cells. However, conventional G-banding cytogenetic analysis showed a normal bone marrow karyotype (Fig. 2). A suspected diagnosis of ALL was made. There was extreme lymphocyte proliferation and the proportion of lymphoblasts was 50.8% (Fig. 1C and D). Flow cytometry (FCM) of bone marrow revealed CD34(+), CD117(−), CD33(+), CD64(−), CD13(+), CD14(−), CD274(−), TSLPR(−), CD11b(−), IgM(+), CD71(−), CD56(+), CD2(−), CD7(−), CD5(−), CD10(+), CD3(−), CD4(−), CD8(−), CD38(+), CD81(+), HLA-DR(+), CD19(+), CD22(+), CD20(+), cMPO(−), cCD3(−), cCD79a(+), TDT(+), CD58(+), CD61(−), CD235a(−), and CD11c(−), and these findings are compatible with BCP-ALL (Fig. 3). Next-generation sequencing (NGS) showed STAG2 gene mutations in the bone marrow, and reverse transcription-polymerase chain reaction (RT–PCR) showed that common fusion genes for BCP-ALL, including *TCF3-PBX1*, *TCF3-HLF*, *ETV6-RUNX1* and *BCR-ABL*, were negative. Other less common fusion genes for Ph-like ALL were also negative.

Her medical history was unremarkable. On physical examination, the patient had an anemic appearance without ecchymosis. The initial laboratory evaluation revealed lymphocytosis (2.37 × 10^9^/l) and moderate anemia (Hb78 g/l, vitamin B12 164 pg/ml, folic acid 3.00 ng/ml and serum ferritin 426.7 ug/l). She was diagnosed with BCP-ALL with involvement of both kidneys.

After a cycle of the VCDLP regimen, the bone marrow was obviously hyperplastic and active, and immature lymphocytes were occasionally observed. FCM showed the ratio of lymphoblasts was 42% with CD34(+), CD10(−), CD19(+), CD38(+), HLA-DR(+), CD64(−), CD13(+), CD20(−), and CD33(+). Since the patient did not achieve remission, a cycle of FLAG salvage treatment was administered, and then the patient was assessed as complete remission (CR). No lymphoblasts were seen in the bone marrow. FCM showed that the ratio of lymphoblasts was 0.8% with CD34(+), CD10(−), CD19(+), CD38(+), HLA-DR(+), CD13(+) and STAG2 gene mutations. The volume of both kidneys returned to normal according to color Doppler ultrasound. After the patient achieved CR, an intrathecal drug injection was performed for consolidation therapy. Eight months after the continued complete remission (CCR), the disease relapsed. A bone marrow smear revealed 55.2% lymphoblasts, and FCM showed ALL with partial expression of CD33. FISH showed *ZNF384* rearrangement (39%) according to its probe (Fig. 1F) and positivity for IgH rearrangement (37%). The leukemia fusion genes and mutation panels were both negative. However, there was no remission in the bone marrow after she was treated with chidamide and a dose-adjusted FLAG plus VP chemotherapeutic regimen, as well as a highly sensitive treatment, HAD. The percentage of lymphoblasts was 14.4% and *ZNF384* rearrangement was positive (17.6%) according to FISH. Currently, the patient has been admitted to the hospital and is receiving chemotherapy regularly. The details of the treatment process are summarized in Additional file 1.

## Discussion and conclusions

Patients harboring *ZNF384* rearrangements are likely to possess the characteristic immunophenotype of a dull or negative CD10 and aberrant expression of one or more myeloid antigens. Thus, *ZNF384*-rearranged ALL is a new subtype of leukemia that may be diagnosed as BCP-ALL [8]. CD10 was negative in our case, while CD13 and CD33 were positive, which is consistent with previous studies. The Tokyo Children’s Cancer Study Group (TCCSG) reported a similar outcome to ours. TCCSG tested 91 samples from BCP-ALL patients, revealing an incidence of a weak or negative expression of CD10, ranging from 0.39 to 67.34% (mean: 19.44 ± 18.23%), and 31.82% and 77.27% expression of CD13 and CD33, respectively, in 22 patients with *ZNF384*-related fusion genes [5].

Unlike the mechanism by which subclonal genomic variation drives clonal evolution in the disease progression of ALL [9], mutational variegation does not determine the immunophenotype for individual patients harboring *ZNF384* rearrangements, and immunophenotypic aberrancy arises from inherent lineage plasticity [8]. Moreover, fusion partners for *ZNF384* are commonly involved in BCP-ALL, which is responsible for leukemia development. Preclinical studies found that the initial effect of fusion proteins was the inhibition of B-cell differentiation by introducing *EP300*, one of the fusion genes, and *ZNF384* into mouse pro-B cells, resulting in the stasis of cell differentiation [10, 11].

The prognosis of *ZNF384* rearrangements in children or adolescents remains unclear. A retrospective study analyzed a total of 218 *ZNF384*-rearranged ALL cases, with ages ranging from 1 to 25. The 5-year event-free survival (EFS) rate was 85% (95% CI 78–90%), and the 5-year overall survival (OS) rate was 91% (95% CI 85–95%) for all patients, suggesting a good outcome [12]. Mary Shago also analyzed a cohort of 240 pediatric patients who were diagnosed with BCP-ALL. Seven of the 240 patients were identified to have ZNF384 rearrangements, with an average age of 5.1 years. The EFS ranges from 6 years 2 months to 9 years 2 months and all patients achieved remission with no relapse [13]. Although a few studies support a generally favorable prognosis in children or young adults with *ZNF384* rearrangements, others suggest a poor clinical outcome. A higher recurrence rate of over 2% was reported in some pediatric *ZNF384* gene fusion cases [5]. Nishimura reported two pediatric *TCF3-ZNF384*-positive patients who developed disease relapse more than 10 years after diagnosis [14]. Additionally, CD10 negativity was found to be an independent adverse prognostic factor in ALL because CD10-negative lymphoblastic leukemia cells had lower cycling capacities and were resistant to apoptosis [15, 16]. A cohort study in China investigating 111 pediatric patients with BCP-ALL revealed that leukemic cells from patients with *ZNF384* fusions were more likely to be CD10-negative than those from other pediatric patients (18.8% vs. 2.9%, *P* = 0.02) (17). This finding may explain why children with *ZNF384* rearrangements have unfavorable outcomes. The adolescent patient in our case had the distinct immunophenotype of CD10 negativity, and she developed disease relapse only eight months after she reached CR despite receiving consistent consolidation chemotherapy. She also had poor responses to multiple chemotherapeutic therapies, necessitating frequent adjustments to more effective and powerful regimens. Our report supplements the unfavorable outcomes of adolescent patients with *ZNF384* rearrangements in the existing literature and supports the view that *ZNF384* rearrangements are correlated with a poor prognosis. However, larger-scale multicenter studies are required to evaluate the exact prognosis of *ZNF384* rearrangements, especially in children or adolescents.

In conclusion, we report a BCP-ALL case with a *ZNF384* rearrangement. The adolescent patient had very rare renal involvement as the first manifestation. However, she showed a very typical immunophenotype of *ZNF384*-rearranged ALL with CD10 negativity and CD13 and CD33 positivity. She responded poorly to chemotherapy and developed a relapse shortly after achieving CR. Her bone marrow karyotype was normal, which may suggest that the *ZNF384* rearrangement-related abnormal karyotype was under the detection limit of conventional G-banding. This indicates that more sensitive methods, such as FISH, should be used. Owing to the low detection rate and variety of treatment protocols from institution to institution, the statistical significance of clinical outcomes in children or adolescents with *ZNF384* rearrangements has not been analyzed thus far. However, it has been linked to an unfavorable prognosis in some small studies, but this still needs to be replicated on a larger scale to be confirmed. Furthermore, *ZNF384* break-apart probes are not commonly included in screening leukemia translocations, which could cause a delay in diagnosis and treatment. Therefore, we suggest that when the rearrangement of *ZNF384* is suspected based on its characteristic immunophenotype, it should be tested and confirmed as soon as possible.

## Supplementary Information

**Additional file 1** The treatment process of the patient.

## Data Availability

All data generated or analysed during this study are included in this published article [and its supplementary information files].

## Abbreviations

ALL: Acute lymphocytic leukemia
BCP-ALL: B-cell precursor acute lymphoblastic leukemia
*ZNF384*: Zinc-finger protein 384
FISH: Fluorescence in situ hybridization
FCM: Flow cytometry
NGS: Next generation sequencing
RT-PCR: Reverse transcription-polymerase chain reaction
CR: Complete remission
CCR: Continued complete remission
TCCSG: The Tokyo Children’s Cancer Study Group
EFS: Event-free survival
OS: Overall survival
