# Supplementary material for: ZNF384 rearrangement in acute lymphocytic leukemia with renal involvement as the first manifestation is associated with a poor prognosis: a case report
Source: Mol Cytogenet. 2022 Feb 14;15:4. doi: 10.1186/s13039-022-00583-4 (PMC8842518; doi:10.1186/s13039-022-00583-4)
Supplement: Supplementary file 1 — Additional file 1 The treatment process of the patient. [file 13039_2022_583_MOESM1_ESM.docx]

**Additional file 1.** The treatment process of the patient

| Date of the treatment | 2020-01-17 | 2020-02-11 | 2020-03-26 | 2020-05-09  2020-06-09 | 2020-08-04 | | 2020-11-20 | | 2020-12-31 |
| --- | --- | --- | --- | --- | --- | --- | --- | --- | --- |
| Regimen | VCDLP 1 cycle | FLAG 1 cycle | CAM 1 cycle | MTX+L-ASP  2 cycles | COATD 3cycles  Consolidation Chemotherapy^*^ 1 cycle | | Chidamide +  adjusted dose of FLAG 2 cycles  VP 2 cycles | | HAD 1 cycle |
| Date of the examination | 2020-02-10 | 2020-03-24 | 2020-05-08 | 2020-07-31 | 2020-09-18 | 2020-11-11 | 2020-12-09 | 2020-12-22 | 2020-02-01 |
| Primary cells proportion of bone marrow smear | 42% | 0.8% | 2.8% |  | 2.8% | 55.2% | 62.0% | 25.6% | 14.4% |
| FCM of primary  cells proportion | 45.21% | 0.36% | 0.27% | 1.2% | 0.29% | 31.6% | 48.91% | 41.9% | - |
|  | CD34(+),CD10(-),CD19(+),CD38(-),HLA-DR(+),CD64(-),CD13(+),CD20(-),CD33(+) | CD34(+),CD10(-),CD19(+),CD38(+),HLA-DR(+), CD13(+) | CD34(+),CD10(-),CD19(+),CD33(+),HLA-DR(+), CD13(+) | - | - | CD33(+) | CD34(+),CD10(+),CD19(+),  CD33(+),CD38(+), CD99(+) | - | - |
| FISH | - | - | - | - | - | *ZNF384* rearrangement(39%)  IgH  rearrangement(37%)  negative for leukemia fusion genes | - | *ZNF384* rearrangement(17.6%)  IgH rearrangement(37%)  negative for leukemia fusion genes | - |

(1)VCDLP regimen: vindesine 4mg on d1,8,15 and 22, cyclophosphamide 0.75g on d1, daunorubicin 60mg on d1and d15,methylprednisolone 60mg every day, and asparaginase 10000IU on d11,14,17,20,23 and 26 of the 28-day cycle; (2)FLAG salvage treatment: fludarabine phosphate 50mg and cytarabine 3000mg from d1to d5 on each 28-day cycle; (3)CAM consolidation chemotherapy :cyclophosphamide 0.75g/m^2^ on d1 and d8, cytarabine 150mg/m^2^ from d1 to d3 and from d8 to d10 and mercaptopurine 60mg/m^2^ from d1 to d7 on each cycle; (4)MTX+L-ASP regimen: methotrexate 5600mg on d1and L-asparaginase10000IU on d3 and d4 of the 28-day cycle;(5)COATD regimen: cyclophosphamide 1.2g on d1, vindesine 4mg on d1, cytarabine 170mg from d1 to d7, etoposide 0.1g from d1 to d4 and dexamethasone 10mg from d1 to d7 on each cycle; consolidation chemotherapy^*^ : cyclophosphamide 1.8g on d1, pegasin 3750IU on d2, mercaptopurine 100mg from d1 to d14, and Arac-C 70mg q12h from d3 to d6 and from d10 to d13 on each cycle; (6)chidamide and adjusted dose of FLAG: chidamide 30mg bid for 4 weeks, fludarabine 50mg and cytarabine 3g from d1 to d5 on each 28-day cycle; VP regimen: vincristine 2mg on d1and d8, and prednisone 70mg from d1 to d14 on each cycle; (7)HAD regimen: homoharringtonine 3.6mg and cytarabine 180mg from d1 to d7, and daunorubicin 70mg from d1 to d3 on each 28-day cycle;

FCM: flow cytometry; FISH: Fluorescence in situ hybridization
